# Electronic Component Mounting for Durable E-Textiles: Direct Soldering of Components onto Textile-Based Deeply Permeated Conductive Patterns

**DOI:** 10.3390/mi11020209

**Published:** 2020-02-18

**Authors:** Tomoya Koshi, Ken-ichi Nomura, Manabu Yoshida

**Affiliations:** Sensing System Research Center (SSRC), National Institute of Advanced Industrial Science and Technology (AIST), 1-1-1 Higashi, Tsukuba, Ibaraki 305-8565, Japan; k-nomura@aist.go.jp (K.-i.N.); yoshida-manabu@aist.go.jp (M.Y.)

**Keywords:** e-textiles, electronic component mounting, printing, soldering, conducive ink

## Abstract

For the improvement of the performance and function of electronic textiles (e-textiles), methods for electronic component mounting of textile circuits with electrical and mechanical durability are necessary. This manuscript presents a component mounting method for durable e-textiles, with a simpler implementation and increased compatibility with conventional electronics manufacturing processes. In this process, conductive patterns are directly formed on a textile by the printing of conductive ink with deep permeation and, then, components are directly soldered on the patterns. The stiffness of patterns is enhanced by the deep permeation, and the enhancement prevents electrical and mechanical breakages due to the stress concentration between the pattern and solder. This allows components to be directly mounting on textile circuits with electrical and mechanical durability. In this study, a chip resistor was soldered on printed patterns with different permeation depths, and the durability of the samples were evaluated by measuring the variation in resistance based on cyclic tensile tests and shear tests. The experiments confirmed that the durability was improved by the deep permeation, and that the samples with solder and deep permeation exhibited superior durability as compared with the samples based on commercially available elastic conductive adhesives for component mounting. In addition, a radio circuit was fabricated on a textile to demonstrate that various types of components can be mounted based on the proposed methods.

## 1. Introduction

In recent years, electronic textiles (e-textiles) have attracted significant research attention with respect to the development of new biological information monitoring systems [1,2,3,4,5,6], human interface systems [7,8], and fashions [9,10], among other schemes [11,12,13,14,15,16,17,18]. At present, only conductive tracks and pads and several types of sensors can be formed on a textile by weaving, knitting, and stitching of conductive yarns on the textile [9,19,20,21], or by printing of conductive inks on the textiles [3,22,23]. Moreover, various types of electronic components such as transistors, batteries, and microprocessors cannot be directly fabricated on a textile by the weaving, knitting, and stitching, as well as printing processes. Therefore, it is necessary to mount the components on the textile circuit, which are fabricated by other processes, to improve the performance and function of e-textiles. In previous studies, to mount the components on textile circuits, the following two types of mounting methods were used: stitching of conductive yarns on the component electrodes [9,19,20,21] and adhesion of the components on the electrodes printed on a textile by conductive adhesives [23,24,25,26]. With respect to previous methods, small electronic components such as surface mount devices cannot be mounted. With respect to the current methods, the mechanical strength of connection points cannot sustain deformations. In addition, the electrical resistance of connection points formed by these methods is higher than that of the soldering method, which is a conventional method for the electronic component mounting of electronics manufacturing. Component mounting methods, which allow for the mounting of various components on textile circuits with electrical and mechanical durability, have not been realized.

The focus of this study is on the direct soldering of components on textile circuits. The direct soldering of components on the conductive patterns printed on a textile achieves a simpler operating process and higher compatibility with conventional electronics manufacturing processes. The materials widely employed for textiles, such as cottons and polyesters, exhibit high heat resistance. This indicates that soldering can be employed for textile circuits with respect to the solution of heat-resistance issues. However, when components are soldered on the patterns on a textile, the patterns are typically peeled off and removed from the textile by solder leaching, and electrical and mechanical breakage occurs. In addition, if the patterns are not peeled off and removed, there is stress concentration at the boundary between the soldered point and the patterns due to the significantly different stiffnesses, and breakage easily occurs due to the deformation of the textile.

To solve these problems, enhancement of the stiffness of conductive patterns printed on a textile by controlling the permeation depth of patterns is proposed in this manuscript. When conductive ink is directly printed on a textile under specific conditions, the ink permeates into the textile and forms thick conductive patterns at a significant depth. The permeation is a unique phenomenon, which allows formation of thicker patterns than the patterns formed on plastic films, as employed for conventional printed electronics. The deep permeation of patterns prevents the peeling and stress concentration by soldering. In addition, the proposed approach is compatible with other durability-enhancement methods, such as the formation of small rigid islands only at the connection point of components [23] and the gradual change in stiffness around the connection point which reduces the stress concentration. To verify the improvement in durability by deep permeation, a type of surface mount component was soldered on printed patterns with different permeation depths, and the durability of the samples was evaluated by measuring the variations in resistance due to mechanical deformation. In this study, cyclic tensile tests and shear tests were conducted under the consideration that e-textiles are typically subjected to the cyclic tensile deformation of the textiles or single excessive shear forces applied to the mounted components. In the experiments, samples were prepared using a commercially available elastic conductive adhesive for component mounting, and the durability of the samples was compared. In addition, a radio circuit was fabricated on a textile to demonstrate that various types of components could be mounted based on the proposed method.

## 2. Materials and Methods

Stretchable silver ink, plain weave cotton, and screen printing were employed as the conductive ink, textile, and printing method in this study. The stretchable silver ink was more applicable than non-stretchable conductive inks, to maintain the conductivity of e-textiles against deformation. In addition, stretchable silver ink has a lower Young’s modulus (68 MPa) than the plain weave cotton used in this study (149 MPa); therefore, the mechanical properties of plain weave cotton, such as the softness and flexibility, are not considerably altered by printing the ink on the textile, even with the deep permeation. As described in Appendix A, we used tensile tests to measure and compare the mechanical properties of plain weave cottons without and with the ink. The plain weave cotton is one of the most frequently used textile materials in the industry, with high heat resistance. Moreover, screen printing is the most frequently used printing process in the industry. In this study, to change the permeation depth of conductive patterns, the ink viscosity and printing conditions were changed. In general, the conductive inks used in screen printing have a high viscosity (greater than 10 Pa·s); therefore, the inks do not have a significant permeation depth. Moreover, conductive ink with low viscosity (less than 10 mPa·s), which is typically used for inkjet printing, exhibits deep permeation. However, in this case, the electrical path is not formed in the printed pattern, given that the conductive particles in the ink are significantly spread and not in contact within the pattern. Therefore, the ink viscosity was set as 1 to 10 Pa·s by diluting the ink with an organic solvent, which is suitable for deep permeation and screen printing. In this study, both undiluted and diluted inks were used. The viscosities of the undiluted and diluted inks were 20.5 Pa·s and 4.6 Pa·s, respectively, at a shear rate of 100 s^−1^. The solder paste and conductive adhesive used in this study were commercially available. The adhesive was an elastic adhesive used on flexible substrates.

**Figure 1:**
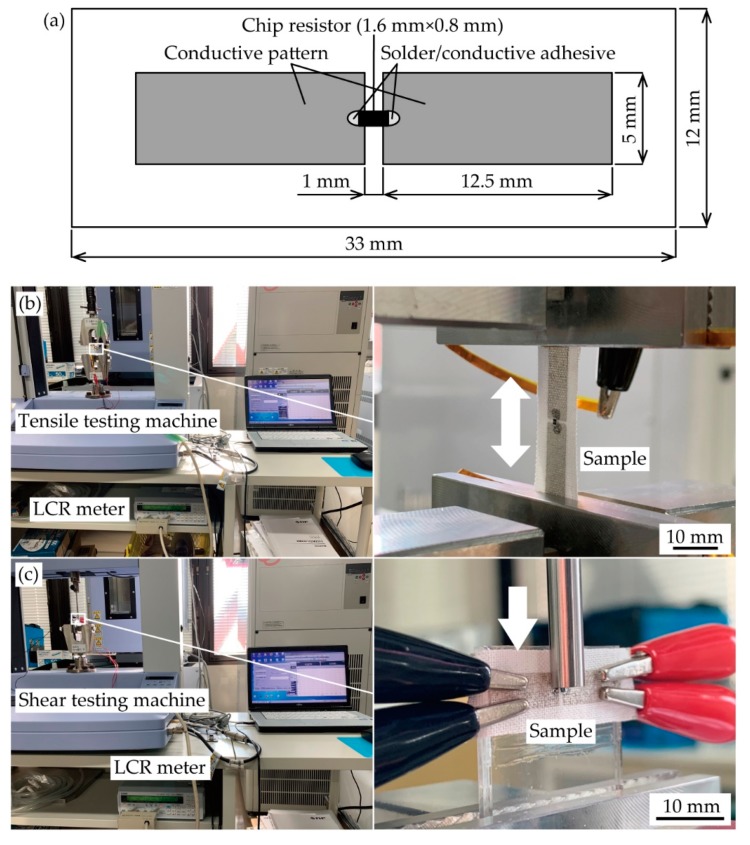
(**a**) Schematic illustration of prepared sample. Images of experimental setups; (**b**) cyclic tensile test; and (**c**) shear test.

The samples were prepared as follows: An undiluted and diluted stretchable silver ink (SSP2801, Toyobo Co., LTD., Osaka, Japan) was printed on a plain weave cotton, which was commercially available, by screen printing. With respect to the textile, the diameter and pitch of the yarns were 0.2 mm and 0.3 mm, respectively, and the textile thickness was 0.22 mm. With respect to the printing conditions, the undiluted ink was screen printed on the textiles once using a screen mask having 200 meshes per inch. For the deeper permeation, the diluted ink was screen printed on the textile three times using a screen mask having 70 meshes per inch. The formed conductive patterns were a pair of electrodes; and the electrode width, length, and gap between the electrode were 5 mm, 12.5 mm, and 1 mm, respectively (Figure 1a). The pattern width was designed to limit the resistance of conductive patterns by applied deformations [27]. After printing, the samples were baked at 100 °C for 30 min and, then, a chip resistor of 0 Ω (RK73Z1JTTD, KOA Co., Nagano, Japan) was mounted on the electrode gap using a solder paste (SMX-H05, Sunhayato Co., Tokyo, Japan) or conductive adhesive (CN-3160L, Kaken Tech Co., Ltd., Osaka, Japan). The chip dimensions were 1.6 mm × 0.8 mm. The mass of solder or conductive adhesive applied to each electrode was approximately 1 mg. The soldering was conducted at 250 °C to 300 °C with an iron, and the conducive adhesive was cured at room temperature for 1 h.

Figure 1b presents the experimental setup for the cyclic tensile test. A prepared sample was mounted on the tensile testing machine (AGS-X, Shimadzu Co., Kyoto, Japan), and cyclic tensile deformation was applied to the sample. The elongation rate and deformation speed rate were 5% and 5 mm/min, respectively. The electrical resistance of the sample was measured with an inductance-capacitor-resistance (LCR) meter (ZM2371, NF Co., Kanagawa, Japan) during the test. The resistance between the pair of electrodes was measured using four probe methods.

Figure 1c presents the experimental setup of the shear test. First, a prepared sample was adhered to the glass plate with an epoxy adhesive. The thickness of the epoxy adhesive layer was sufficiently thin, and therefore it did not have an influence on the adhesion between the chip resistor and textile. The glass plate with the sample was mounted on the shear testing machine (AGS-X, Shimadzu Co., Kyoto, Japan), and shear deformation was directly applied to the resistor by a jig. The deformation displacement and deformation speed rate were 0.4 mm and 0.5 mm/min, respectively. In addition, the resistance of the sample was measured using an LCR meter during the test, based on four probe methods.

## 3. Results and Discussion

### 3.1. Observation of Printed Conductive Patterns and Mounted Electronic Components before the Test

**Figure 2:**
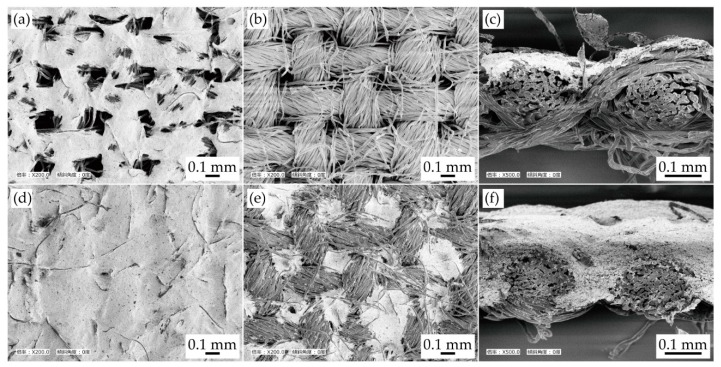
SEM (scanning electron microscope) images of conductive patterns formed on a plain weave cotton, prior to the test. SEM image of (**a**) printing side; (**b**) alternate side; and (**c**) cross-section of less permeated pattern. SEM image of (**d**) printing side; (**e**) alternate side; and (**f**) cross-section of deeply permeated pattern.

Figure 2 presents the scanning electron microscope (SEM) images of the conductive pattern formed on a plain weave cotton with different permeation depths, prior to the test. The patterns with lower and greater permeation depths are shown in Figure 2a–c and Figure 2d–f, respectively. On the one hand, the patterns with lower permeation depths were only formed on the printed side of each yarn; thus, the pattern formed a mesh-like structure. On the other hand, as shown in Figure 2d–f, the conductive patterns with deeper permeation formed a thicker film-like structure, and the printed ink was permeated in the gap between the yarns.

The effective thickness of the conductive pattern was calculated from the changes in the mass of the samples and density of silver ink, given that the thickness was not uniform; and it was difficult to measure the thickness from the cross-sectional images, as shown in Figure 2c,f. The calculation was carried out with respect to the following samples: three types of strip-shaped samples, a plain weave cotton, a plain weave cotton with less permeated ink, and a plain weave cotton with deeply permeated ink. The materials and printing conditions were the same as those described above. The width and length of the samples were 20 mm and 60 mm, respectively. The mass of each sample was measured, and the mass of the printed ink was obtained by subtracting the mass of the plain weave cotton from that of the sample with ink. The volume of printed ink was calculated by dividing the mass of the printed ink by the density of the ink (5.0 × 10^3^ kg/m^3^). The average thickness was calculated by dividing the volume by the width and length of the sample. As a result, the effective thickness of the less and deeply permeated patterns were 9 µm and 50 µm, respectively. Hence, in particular, the effective elongating stiffness (the product of the Young’s modulus and cross-sectional area) of the deeply permeated pattern was greater than that of the less permeated pattern by a factor of five.

**Figure 3:**
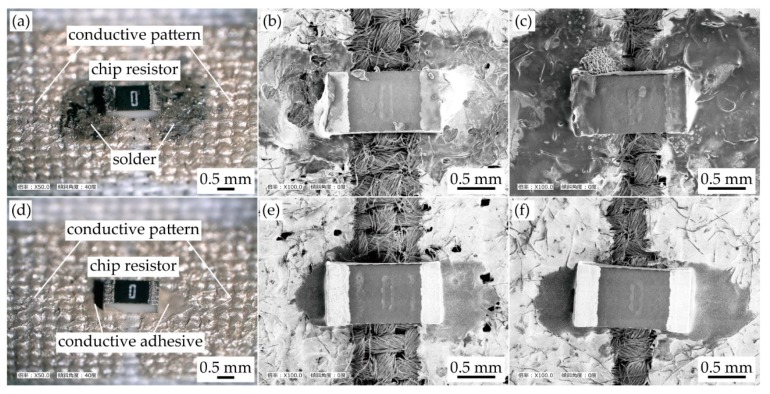
Optical and SEM images of prepared samples prior to the test. (**a**) Optical image of soldered chip resistor on conductive pattern formed on a plain weave cotton; SEM images of samples with solder and patterns at (**b**) lower; and (**c**) greater permeation depth; (**d**) optical image of chip resistor using conductive adhesive; SEM images of samples with conductive adhesive and patterns at (**e**) lower; and (**f**) greater permeation depths.

Figure 3 presents the optical and SEM images of a chip resistor mounted on conductive patterns, prior to the test. The chip resistors mounted with solder and conductive adhesive are shown in Figure 3a–c and Figure 3d–f, respectively. Figure 3a–c confirms that the chip resistor was mounted with solder spreading on the conductive pattern. It was confirmed that a portion of the less permeated pattern was peeled off and removed by soldering, as shown in Figure 3b. Moreover, it was confirmed that all of the prepared samples with solder or conductive adhesive had electrical and mechanical connections between the chip resistor and pattern.

### 3.2. Cyclic Tensile Test

**Figure 4:**
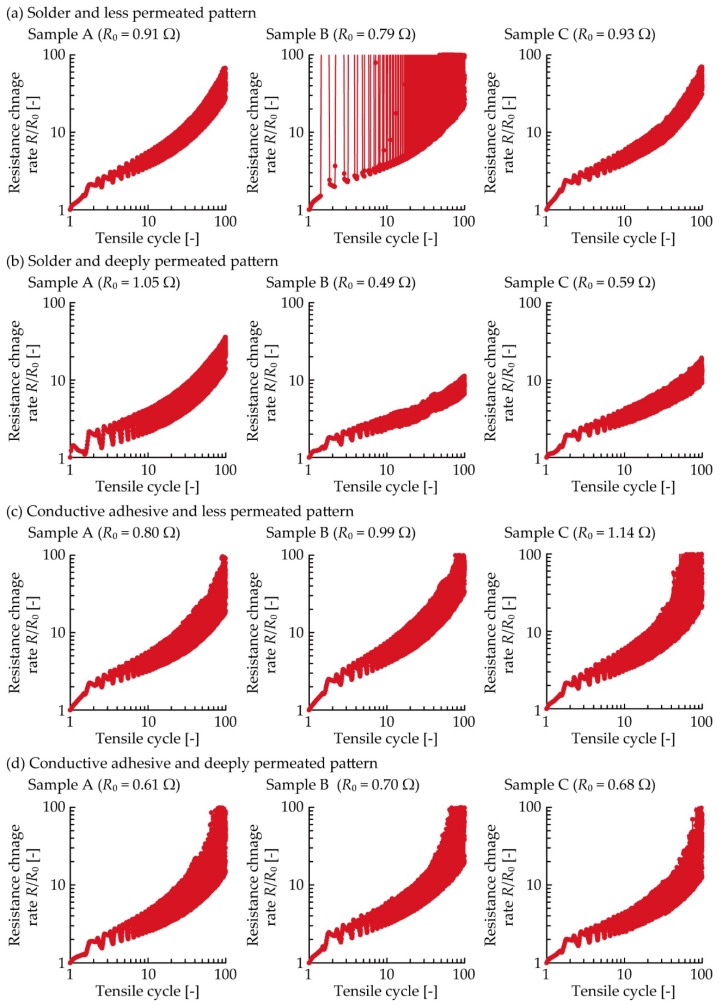
Resistance variation under 5% cyclic tensile deformation. Samples with solder and patterns at (**a**) lower; and (**b**) greater permeation depths; and with conducive adhesive and patterns at (**c**) lower; and (**d**) greater permeation depths.

Figure 4 presents the variation in resistance of each sample under a 5% cyclic tensile deformation. The resistance change rate was calculated by dividing the resistance *R* by the initial resistance *R*_0_. Three samples (A, B, and C) were tested under the following conditions: (i) soldering with less permeated pattern, (ii) soldering with deeply permeated pattern, (iii) conductive adhesive with less permeated pattern, and (iv) conductive adhesive with deeply permeated pattern. Figure 4a presents the results for the samples with the solder and less permeated pattern. The resistance increased and decreased repeatedly during each tensile cycle, and it gradually increased in accordance with an increase in the cycle number. In particular, Sample B exhibited a higher resistance change rate (greater than 100) during and after the first cycle. Figure 4b presents the results of the samples with the solder and deeply permeated pattern, and the resistance was found to increase, as shown in Figure 4a. A comparison with Figure 4a,b, confirms that the resistance change rate with respect to the tensile cycle was smaller. With respect to the samples wherein a conductive adhesive was used (Figure 4c,d), the resistance increased, similar to that of the samples where solder was used (Figure 4a,b). However, the range of the resistance change rate was wider than that of the samples with solder, and the resistance change rate around the 100th cycle was greater than 100. 

**Figure 5:**
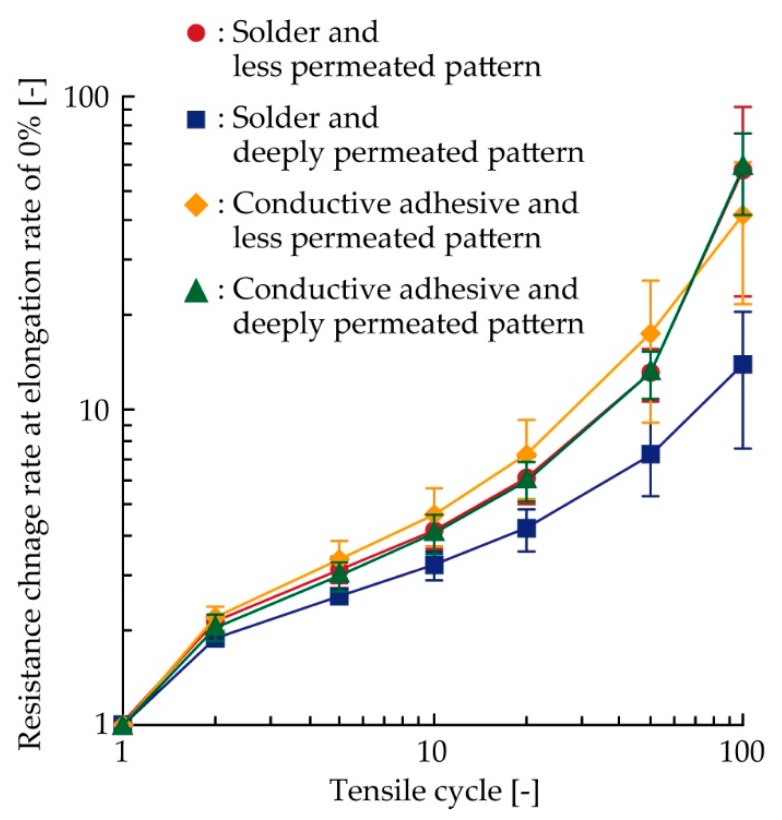
Integrated plots of the resistance variation shown in Figure 4.

For the numerical comparison between each condition shown in Figure 4, all the plots were integrated into a single graph, as shown in Figure 5. For clarity, the average resistance change rates of the three samples at a 0% in elongation rate during the first, second, fifth, 10th, 20th, 50th, and 100th cycles are shown. The average resistance change rate at each condition increased as the number of tensile cycles increased. Comparing the plots for the soldered samples with the less and deeply permeated patterns, the average resistance change rate at the 100th cycle was decreased from 58 to 14 owing to the deep permeation. This indicates that the durability against cyclic tensile deformation was improved by controlling the permeation depth of the conductive pattern. The plots for the sample with the conductive adhesive with the less and deeply permeated pattern show average resistance change rates of 42 and 59, respectively, at the 100th cycle. These values are 3.0 to 4.2 times greater than that of the soldered sample with the deeply permeated pattern. This indicates that the soldered samples with the deeply permeated pattern exhibited greater durability against the cyclic tensile deformation as compared with the samples using the conductive adhesive.

**Figure 6:**
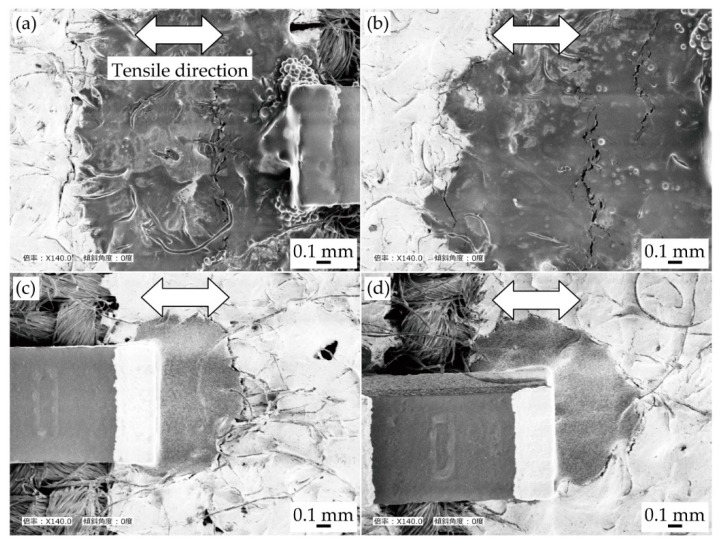
SEM images around connection point of chip resistor after cyclic tensile test. Samples with solder and patterns at (**a**) lower; and (**b**) greater permeation depths; and with conductive adhesive and patterns at (**c**) lower; and (**d**) greater permeation depths.

Figure 6 presents the SEM images around the connection point of the chip resistor after the cyclic tensile test. Under each condition, crack initiations and propagations were observed at the boundary between the solder/conductive adhesive and the pattern. This indicates that the stress concentration at the boundary between the solder/adhesive and pattern was larger than that at the other connection point, i.e., the boundary between the solder/adhesive and component. The increase in resistance shown in Figure 4 and Figure 5 are assumed to be due to the crack initiations and propagations at the boundary between the solder/adhesive and the pattern. Therefore, this also indicates that controlling the permeation depth was effective in reducing the increase in resistance due to the cyclic tensile deformation. However, compared with the samples using the conductive adhesive and the less and deeply permeated patterns, the differences due to the control of the permeation depth were not significant, as shown in Figure 4 and Figure 5. In this study, the Young’s modulus values of the stretchable silver ink (68 MPa) and the elastic conductive adhesive (73 MPa) were similar. The stress concentration between the conductive adhesive and the pattern must be lower than that between the solder and the pattern (the Young’s modulus of the solder was 22 GPa). Therefore, there was a lower reduction in the stress concentration, and the differences due to the control of the permeation depth were not significant, in the case of conductive adhesive.

### 3.3. Shear Test

**Figure 7:**
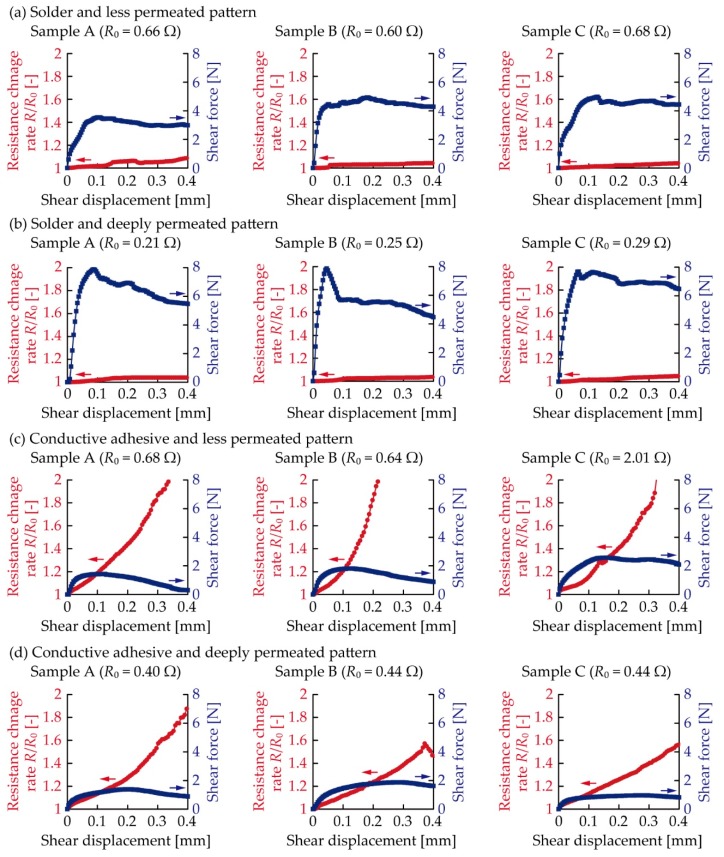
Resistance and shear force variation in shear force test. Samples with solder and patterns at (**a**) lower; and (**b**) greater permeation depths; and conductive adhesive and patterns at (**c**) lower; and (**d**) greater permeation depths.

Figure 7 presents the resistance and shear force variation of each sample due to shear deformation. The resistance change rate was calculated by *R*/*R*_0_, and three samples (A, B, C) were tested under the four conditions as with the cyclic tensile test. Figure 7a presents the results for the samples with solder and patterns at a lower permeation depth. The resistance gradually increased in accordance with an increase in the shear displacement. Moreover, the shear force abruptly increased when the shear displacement was lower and, then, gradually decreased in accordance with an increase in the shear displacement. Figure 7b presents the results of the samples with the solder and deeply permeated patterns. The resistance gradually increased, as shown in Figure 7a. Moreover, the shear force abruptly increased and, then, gradually decreased, as shown in Figure 7a. However, the maximum value of the shear force, i.e., the peak shear force, was greater than that of the samples with the less permeated pattern. Figure 7c,d presents the results for the samples with the conductive adhesive. The resistance gradually increased in accordance with an increase in the shear displacement; however, the variation of the rate of change in resistance with respect to the shear displacement was significantly greater than that of the samples with solder. Moreover, the shear force gradually increased and, then, gradually decreased in accordance with a decrease in the shear displacement. The peak shear force was lower than that of the samples with solder.

**Figure 8:**
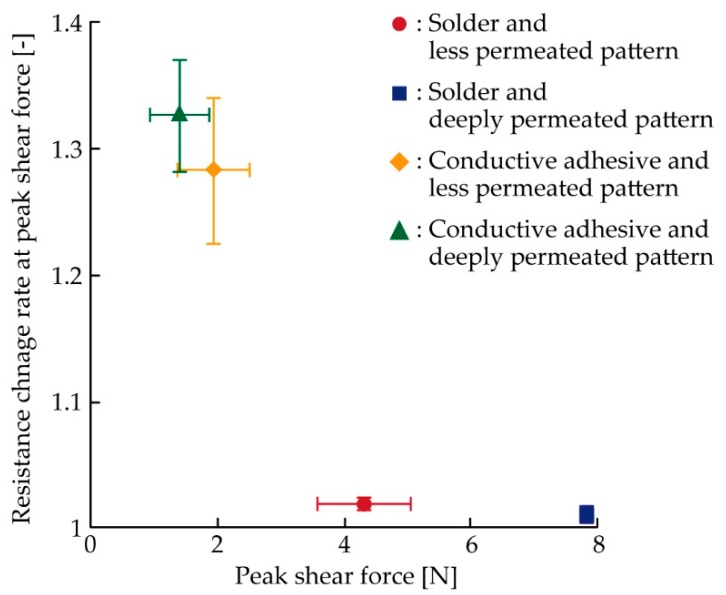
Relationship between peak shear force and resistance change rate at peak shear force.

As shown in Figure 7, the shear displacement at peak deformation was different for each sample, and the resistance change rate varied with respect to the shear displacement at peak deformation. In this case, the effect of the permeation depth control was not clear. Hence, for a clearer understanding of the effect and for the numerical comparison, the relationship between the peak shear force and resistance change rate at the peak shear force was analyzed, as shown in Figure 8. The plots show the average values for the three samples. As can be seen from the figure, the plots at the lower right region represent the electrical and mechanical durability against the shear deformation. Figure 8 confirms that the samples with the solder and deeply permeated pattern exhibited the greatest durability among all the samples. Among the soldered samples, the resistance change rate at the peak shear force was almost the same for the less permeated (1.02) and deeply permeated (1.01) patterns; however, the peak shear force increased from 4.3 N to 7.8 N. This indicates that the mechanical durability was effectively improved by the control of the permeation depth. Regarding the samples using the conductive adhesive, the values of resistance change rate were 1.28 at 1.9 N for the less permeated pattern, and 1.33 at 1.4 N for the deeply permeated pattern. Therefore, the soldered samples with the deeply permeated pattern exhibited a 1.3 times smaller resistance change and a 4.1 to 5.6 times greater peak shear force than the samples using the conductive adhesive. This indicates that the soldered samples with the deeply permeated pattern exhibited greater electrical and mechanical durability against the single shear deformation than the samples using the conductive adhesive did. Moreover, with respect to the samples with the conductive adhesive, the differences between the patterns with the lower and greater permeation depths were not significant. This is also because the Young’s modulus values of the stretchable silver ink and elastic conductive adhesive were similar; thus, there was a lower decrease in the stress concentration around the connection point than in the case wherein the solder was used.

**Figure 9:**
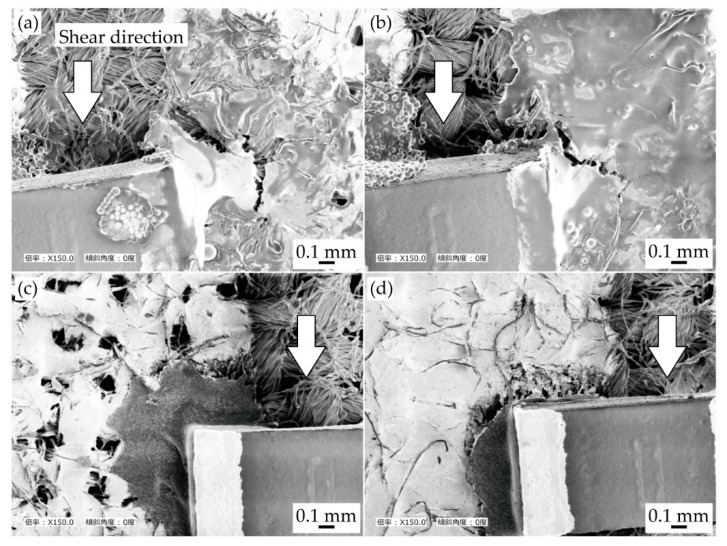
SEM images around connection point of chip resistor after shear test. Samples with solder and patterns with (**a**) lower; and (**b**) greater permeation depths; and with conductive adhesive and patterns with (**c**) lower; and (**d**) greater permeation depths.

Figure 9 presents the SEM images around the connection point of the chip resistor after the shear test. With respect to the samples with solder (Figure 9a,b), the crack propagation and peeling off the conductive pattern were observed around the edge of chip resistor. This indicates that the stress concentration at the edge of chip resistor was larger than the stress concentration at the other connection point, i.e., the boundary between the pattern and solder. Moreover, with respect to the samples with conductive adhesives, crack propagation was observed at the boundary between the conductive adhesive and patterns. This indicates that the stress concentration at the boundary between the pattern and adhesive was larger than the stress concentration at the other connection point.

### 3.4. Fabrication of Radio Circuit on Textile

**Figure 10:**
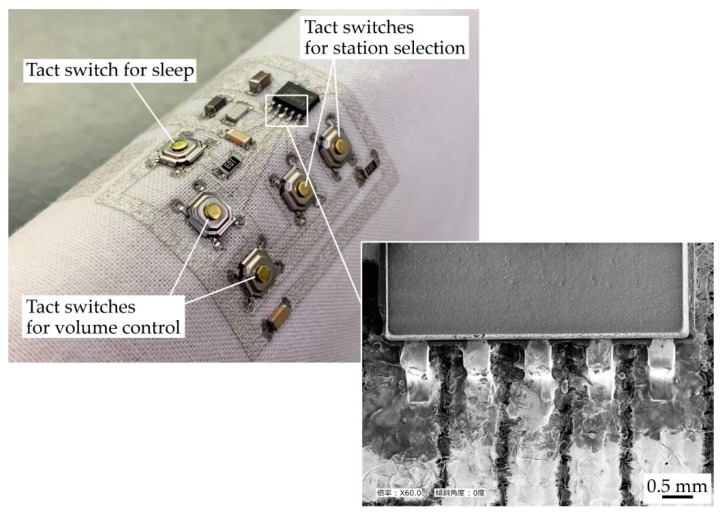
Optical and SEM images of radio circuit fabricated on plain weave cotton.

Figure 10 presents the optical and SEM images of a radio circuit fabricated on plain weave cotton. The fabrication process was the same as that in the fabrication process of the samples with solder and deeply permeated patterns, as described in the previous section. The radio circuit consisted of chip resistors, capacitors, inductors, tact switches, and a microprocessor, among other components. The tact switches were connected to the microprocessor, and station selection, sound volume, and sleep could be controlled by operating the switches. In particular, the SEM image in Figure 10 confirms that the microprocessor with a pin pitch of 1 mm was successfully mounted on the conductive patterns. Moreover, it was confirmed that the radio circuit was driven, especially when the bending deformation was applied to the radio circuit, as shown in Figure 10. This deformation, which had a radius of approximately 20 mm, did not degrade any aspect of the performance, such as the sound volume and noise generation, of the radio circuit. The results confirmed that various types of electronic components, which could not be mounted by conventional mounting methods for e-textiles, were successfully mounted using the proposed method.

## 4. Conclusions

In this paper, an electronic component mounting method for the realization of durable e-textiles was developed. In this method, a conductive ink is printed on a textile with deep permeation of the ink by screen printing; thus, deeply permeated conductive patterns are formed, and the electric components are directly soldered on the patterns. The deep permeation of ink enhances the stiffness of conductive patterns, which prevents electrical and mechanical breakage due to the stress concentration between the conductive pattern and solder. To verify the improvement in durability by deep permeation, a cyclic tensile test and shear test were conducted. As a result, compared with samples using a conductive adhesive, soldered samples with a deeply permeated pattern showed 3.0 to 4.2 times lower changes in resistance in the 100 cyclic tensile test; these samples also showed a 1.3 times smaller resistance change and a 4.1 to 5.6 times greater peak shear force in the shear test. The results confirmed the improvement in durability, and that the samples prepared using the proposed method were electrically and mechanically more durable than the samples prepared using a commercially available elastic conductive adhesive for component mounting. In addition, the radio circuit was fabricated on a textile, which confirms that it can be employed for the mounting of various types of electronic components, i.e., a microprocessor with a pin pitch of 1 mm with high durability, as achieved in this study.

## 5. Patents

T.K., K.-i.N., and M.Y. are declared as inventors on a patent application (Japanese Patent Application no. 2020-010059) resulting from this study.

## Figures and Tables

**Figure A1:**
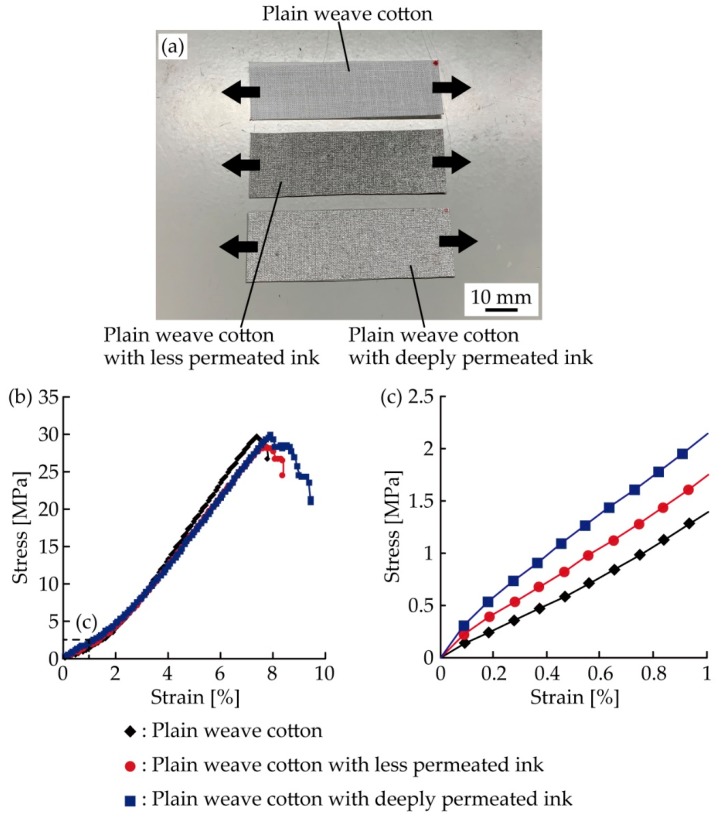
Mechanical property measurement of plain weave cotton without/with less and deeply permeated ink. (**a**) Optical image of samples; (**b**,**c**) Stress-strain curves.

## Appendix A

The mechanical properties of the plain weave cotton without and with the silver stretchable ink were measured through the tensile test. Figure A1a shows an optical image of the samples used in the test. Three types of samples were prepared as follows: plain weave cotton, plain weave cotton with less permeated ink, and plain weave cotton with deeply permeated ink. The samples with the less and deeply permeated inks were prepared using the same procedures as described in Section 2. The dimensions of the samples were 20 mm × 60 mm. Each sample was mounted on the tensile testing machine (AGS-X, Shimadzu Co., Kyoto, Japan), and a tensile deformation was applied to the sample at deformation speed rate of 5 mm/min. Figure A1b,c shows the stress–strain curve for each sample. When the strain was low (around 1%, as shown in Figure A1c), the stress in the samples without the ink and with the less and deeply permeated ink increased to 1.4 MPa, 1.7 MPa, and 2.1 MPa, respectively. This indicates that printing the ink increased the stiffness of the plain weave cotton by 1.2 times or 1.5 times. However, when the strain increased to around 3% to 4%, the stiffness remained almost the same. These results suggest that the mechanical properties of plain weave cotton are not considerably altered through the printing of silver stretchable ink.
